# Epidemiology of West Nile Virus in New York City: Trends and Transmission Dynamics (2000–2019)

**DOI:** 10.3390/pathogens14040364

**Published:** 2025-04-08

**Authors:** Waheed I. Bajwa, Liyang Zhou

**Affiliations:** Department of Health and Mental Hygiene, New York City, 125 Worth Street, Manhattan, NY 10013, USA

**Keywords:** West Nile virus, epidemiology, transmission dynamics, *Culex pipiens*, *Culex restuans*, *Culex salinarius*

## Abstract

The 1999 outbreak of West Nile virus (WNV) in New York City (NYC) marked the first documented introduction of the virus into the western hemisphere, prompting extensive public health surveillance. This study examines the epidemiology of WNV from 2000 to 2019, analyzing 381 human cases, including 66 cases of West Nile Fever (WNF) and 315 cases of West Nile Neuroinvasive Disease (WNND), with 35 fatalities. Simultaneously, 6632 WNV-positive mosquito pools were identified across 16 species. While *Culex pipiens* and *Cx. restuans* accounted for 91.4% of positive pools, *Cx. salinarius*, which comprised only 6.2%, exhibited a stronger correlation with human infections. Human surveillance involved comprehensive case investigations following laboratory-confirmed WNV infections, incorporating structured interviews with patients and healthcare providers. Mosquito surveillance was conducted through weekly collections from 52–71 permanent trap sites, supplemented by approximately 200 additional sites annually in areas with elevated WNV activity. Captured mosquitoes were species-identified, pooled, and tested for WNV RNA via RT-PCR. Findings highlight the dominant role of *Culex* species, particularly *Cx. salinarius*, in human WNV transmission, with 69% of cases occurring near WNV-positive mosquito pools. Spatial analyses identified transmission hotspots, emphasizing the importance of species-specific mosquito control strategies. Over the study period, WNV activity has increased in NYC, likely influenced by climate change, as warmer summers and extended breeding seasons align with peak outbreaks. Integrating spatial mapping, climate forecasting, and targeted surveillance could significantly improve WNV mitigation efforts in urban environments.

## 1. Introduction

The 1999 West Nile virus (WNV) outbreak in New York City marked the virus’s first introduction into the western hemisphere, leading to its rapid spread across North America [1,2,3]. Transmission dynamics have been influenced by factors such as climate change, urbanization, and habitat modifications affecting mosquito populations [4,5,6,7]. Rising temperatures and urban heat islands may be extending mosquito breeding seasons, thereby increasing WNV transmission rates [8]. Additionally, genetic studies suggest that ongoing viral evolution could impact mosquito infectivity and disease severity [9]. Given its significant public health impact—resulting in thousands of cases and hundreds of fatalities [3]—WNV has been extensively studied [4,10]. Beyond mosquito bites, transmission has also been reported through blood transfusions and organ transplants, underscoring the need for continued surveillance [11].

Initially, dead bird reports served as early indicators of WNV activity [12]. However, due to logistical challenges such as delays and costs, many states transitioned to mosquito surveillance, with a particular focus on feeding behavior, as a more effective early detection method [4,12]. Despite extensive research examining climatic and landscape factors influencing WNV incidence, consistent patterns remain difficult to establish due to geographic and temporal variations in vector ecology [13,14,15,16,17].

From 1999 to 2019, the New York City Department of Health and Mental Hygiene (DOHMH) conducted extensive WNV surveillance, monitoring human cases, mosquito populations, and environmental conditions affecting transmission [18]. Given the persistent public health burden of WNV, continued epidemiological assessments remain essential. This study synthesizes two decades of data to analyze WNV epidemiology in an urban setting, with a focus on mosquito species dynamics and disease ecology [19,20,21,22].

## 2. Materials and Methods

### 2.1. Study Area

This study was conducted in New York City (NYC), a densely populated urban environment comprising five boroughs: Manhattan, Brooklyn, Queens, the Bronx, and Staten Island. With a population exceeding 8 million, NYC provides a unique setting for studying urban mosquito-borne disease dynamics due to its diverse landscapes, including highly urbanized areas, parks, wetlands, and stormwater retention basins. These varied habitats support multiple mosquito species that play a role in the transmission of West Nile virus.

### 2.2. Surveillance and Data Collection

Following the 1999 WNV outbreak, the DOHMH established an extensive mosquito surveillance program across the city’s five boroughs. Between 2000 and 2019, 381 human WNV cases (both WNV neuroinvasive and fever cases) were reported, along with 6632 positive mosquito pools.

In the U.S., WNV disease is a nationally notifiable condition with standardized surveillance case definitions [23]. For human surveillance, upon receiving electronic laboratory reports from healthcare providers indicating a current WNV infection, the DOHMH initiated comprehensive investigations. This process included structured interviews with healthcare providers and patients or their proxies to collect detailed information on clinical symptoms, potential risk factors, travel history, and recent exposure to mosquitoes or outdoor environments.

All confirmed human cases underwent plaque reduction neutralization testing (PRNT) to verify WNV infection, in accordance with CDC protocols [23].

Mosquito testing was conducted by the DOHMH Public Health Laboratory, ensuring accurate detection and monitoring of WNV activity across the city.

As recommended by the CDC [23,24] and supported by serosurveys conducted in Queens (1999) and Staten Island (2000), which estimated that for every reported neuroinvasive case there are approximately 30 cases of West Nile fever and 140 subclinical infections [25], the analysis in this study emphasizes WNND cases due to their higher reporting reliability and epidemiological consistency. This approach helps minimize bias introduced by underreporting of mild or asymptomatic infections, which is common in WNF cases.

### 2.3. Mosquito Sampling and Identification

From May to October, mosquitoes were collected weekly from 52–71 permanent trap sites, supplemented by approximately 200 additional sites annually in areas where WNV-positive mosquito pools had been detected. While permanent sites remained active throughout the mosquito season, supplemental sites were used temporarily. Various trapping methods, including carbon dioxide-baited light traps and gravid traps, were strategically placed in areas with high mosquito activity.

Captured mosquitoes were identified to species, and up to 50 individuals of the same species from a single site were pooled for WNV testing. The pooled samples were homogenized to release viral particles, followed by total nucleic acid extraction using the KingFisher Flex system. Extracted nucleic acid was then transferred to a PCR plate for amplification on a QuantStudio instrument, and the presence of WNV RNA was detected using reverse transcription-polymerase chain reaction (RT-PCR).

### 2.4. Statistical and Spatial Analysis

To assess the correlation between human WNV cases and infected mosquito pools, Pearson correlation coefficients were calculated using SAS Enterprise Guide 7.1, with statistical significance set at *p* < 0.05 [26]. This analysis focused exclusively on neuroinvasive disease cases (WNND), as they are more frequently and reliably reported due to the severity of symptoms and hospitalization requirements [18]. WNF cases, by contrast, are frequently underreported because they often present with mild symptoms, leading to lower clinical detection rates. This surveillance bias is well documented in CDC guidance [24] and supported by local serosurveys [25]. Cases with incomplete data (e.g., missing onset dates or addresses) were excluded from the statistical analysis.

Additional analyses examined *Cx. pipiens*, *Cx. restuans*, and *Cx. salinarius* pools collected within a 2-mile radius of human cases (extended to 5 miles for *Cx. salinarius*) within 10 days of symptom onset [27]. Pools from less abundant mosquito species were also cross-referenced with human cases occurring within the same timeframe.

Geographic Information System (GIS) tools (ArcMap v10.6.1) were employed for spatial analysis, identifying high-risk transmission zones through kernel density estimation. Flight range parameters for mosquito species were based on those documented in the literature [28].

### 2.5. Ethical Considerations

This study was conducted as part of routine public health surveillance by the DOHMH. All patient data were anonymized before analysis. Ethical review and informed consent were not required, as the study involved retrospective analysis of de-identified public health surveillance data.

## 3. Results

Between 2000 and 2019, the DOHMH recorded 381 human cases of WNV, including 66 cases of West Nile Fever and 315 cases of West Nile Neuroinvasive Disease, resulting in 35 fatalities. During the same period, mosquito surveillance detected 6632 WNV-positive pools out of 108,071 tested. *Culex pipiens* and *Cx. restuans* accounted for 91.36% of positive samples, while 6.12% were *Cx. Salinarius* (Table 1). Despite extensive collections of other species—including *Aedes albopictus*, *Ae. taeniorhynchus*, *Ae. triseriatus*, *Ae. trivittatus*, *Ae. vexans*, *Anopheles quadrimaculatus*, and *Coquillettidia perturbans*—these species collectively accounted for only 2.52% of WNV-positive pools (Table 1).

Pearson correlation analyses established a statistically significant association between human WNV cases and the presence of infected mosquito pools (r = 0.22, *p* < 0.05). Of the total positive mosquito pools, 986 were linked to human infections: 708 were linked to *Cx. pipiens* and *Cx. restuans*, 251 to *Cx. salinarius*, and 27 to other species (Table 1 and Table 2). Despite being less abundant, *Cx. salinarius* showed a disproportionately strong correlation with human infections, contributing to 60.9% of human-associated mosquito pools, compared to 11.7% for *Cx. pipiens* and *Cx. restuans* (Table 2).

**Figure 1:**
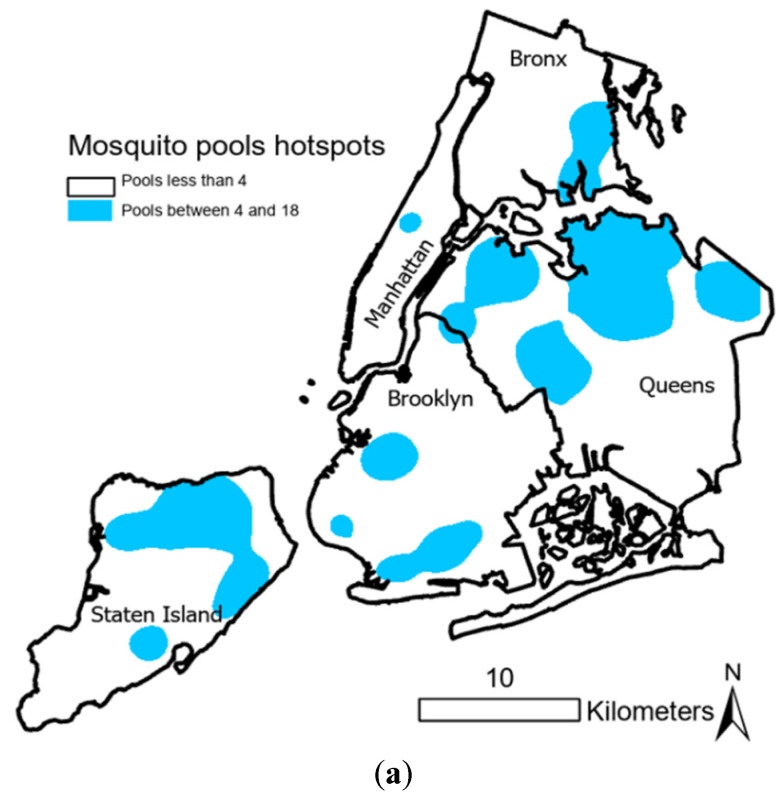

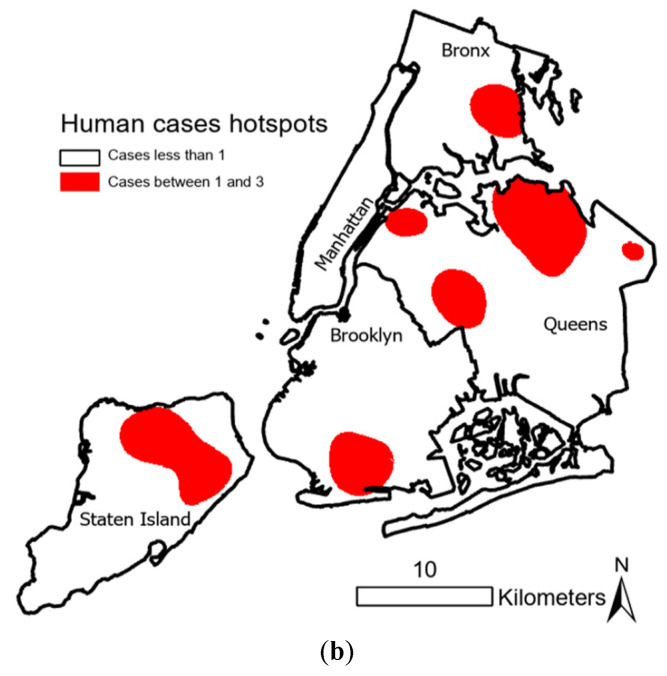
Hotspots of WNV human cases with positive mosquito pools within 10 days before the onset of human disease in NYC, 2000–2019. (**a**) Mosquito pool hotspots. (**b**) Human case hotspots.

Further spatial analysis identified persistent WNV transmission hotspots in northern Staten Island, southern Brooklyn, northwestern and northeastern Queens, and parts of the eastern Bronx (Figure 1). These regions exhibited significant overlap between human cases and positive mosquito pools, highlighting the need for targeted vector control strategies in high-risk urban zones.

Pearson correlation coefficients for each mosquito species further reinforced these findings, with values of 0.87 for *Cx. pipiens* and *Cx. restuans*, 0.78 for *Cx. salinarius,* and 0.89 when considering all positive mosquito species combined. While *Cx. pipiens* and *Cx. restuans* were found to be the dominant vectors due to their prevalence in WNV-positive pools, the data suggest that *Cx. salinarius* plays a critical role in transmission despite its lower numbers.

## 4. Discussion

*Culex* species in New York City exhibit distinct behavioral adaptations compared to their counterparts in rural and suburban areas, largely influenced by urban ecological factors such as limited wildlife hosts, high human density, and fragmented green spaces. In NYC, *Cx. pipiens* and *Cx. restuans* show significant anthropophilic tendencies, with over 25% of their blood meals sourced from humans [18]. This contrasts with nonurban environments, where these species primarily feed on avian hosts, sustaining the WNV enzootic cycle [19,29,30,31,32].

Among the *Culex* species in NYC, *Cx. salinarius* stands out due to its even greater preference for human hosts—approximately 30% higher than *Cx. pipiens* and *Cx. restuans* combined [18]. Despite its lower abundance, *Cx. salinarius* plays a critical role in WNV transmission, acting as a bridge vector between birds and humans. This species’ heightened anthropophilic behavior increases the likelihood of spillover events and urban outbreaks [6,29,30,31,32].

Spatial analyses and blood meal studies confirm *Cx. salinarius* as disproportionately responsible for human infections, despite representing only 6.12% of all WNV-positive mosquito pools. Notably, it accounted for 60.92% of pools associated with human cases, whereas *Cx. pipiens* and *Cx. restuans* contributed just 11.69% (see Table 2). These findings align with recent research by Uelmen et al. (2023), reinforcing the idea that *Cx. salinarius* plays a more substantial role in urban transmission than previously recognized [33].

While *Cx. pipiens* and *Cx. restuans* exhibited a strong correlation with human cases (Pearson correlation coefficient: 0.87, *p* < 0.01), largely due to their dominance in WNV-positive pools (91.4% of total pools) and widespread presence in NYC [1], *Cx. salinarius* displayed a more localized but equally significant correlation (Pearson correlation coefficient: 0.78, *p* < 0.01). This relationship remained evident even when restricting analysis to pools collected within species-specific flight ranges (approximately 5 miles for *Cx. salinarius*) and within 10 days preceding human case onset. These findings highlight the species’ unique role as a high-risk vector [34,35], particularly in dense urban areas with limited avian hosts [18].

Persistent transmission hotspots in northern Staten Island, southern Brooklyn, northeastern Queens, northwestern Queens, and parts of the eastern Bronx (Figure 1) further emphasize the need for targeted interventions. These areas provide favorable breeding conditions, such as stormwater retention basins, and proximity to green spaces or wetlands supporting large mosquito populations. High densities of *Cx. pipiens* and *Cx. restuans* contribute to sustained transmission cycles, while the emergence of *Cx. salinarius* in these hotspots demonstrates its adaptive feeding behavior in urban landscapes [29,30].

In peak years like 2018, mosquito pools reached a record high of 1024, while human WNV cases totaled 36. That year, hotspot areas in Queens and Staten Island exhibited significant overlap between human cases and mosquito activity, underscoring the importance of focusing vector control efforts in these locations. Across the study period (2000–2019), a total of 986 mosquito pools were associated with human cases, including 708 pools of *Cx*. *pipiens* and *Cx*. *restuans*, 251 pools of *Cx. salinarius*, and 27 pools of other species. These results suggest that vector surveillance should not only monitor mosquito abundance but also track species composition, as *Cx*. *salinarius* poses a disproportionate risk for human infections.

These findings carry important implications for public health strategies. The strong human-feeding preference of *Cx. salinarius*, combined with its presence in high-risk urban zones, suggests that control efforts should specifically target this species. Integrating spatial mapping with biological data can help optimize vector control interventions, particularly in hotspot areas during peak transmission periods [29,30]. The ability of *Cx. pipiens* and *Cx. restuans* to thrive in urban environments further reinforces the need for long-term surveillance and environmental modifications, such as eliminating standing water in stormwater basins and maintaining green spaces, which often serve as breeding sites [18,31,36].

Despite these insights, further research is needed to explore the ecological and behavioral adaptations of urban *Culex* populations. Investigating seasonal shifts in host preference, overwintering behavior, and species-specific breeding patterns could provide a deeper understanding of their role in disease transmission. Additionally, examining the effects of climate variability and anthropogenic landscape changes on mosquito populations will be crucial for developing long-term strategies to mitigate WNV and other vector-borne diseases [15,35].

Given the persistent nature of WNV transmission in NYC, Integrated Vector Management (IVM) programs must be adapted to urban ecological conditions. Strengthening public health infrastructure, enhancing mosquito surveillance, and fostering community engagement will be critical in mitigating WNV risks and addressing the emergence of other mosquito-borne diseases such as chikungunya, dengue, and Zika [36].

The record-high WNV activity observed in NYC in 2018—nearly two decades after its initial emergence—suggests that climate change, including prolonged mosquito breeding seasons and urban heat island effects, is intensifying transmission risks. Rising temperatures and increased heat retention in cities create optimal conditions for mosquito survival and viral amplification, extending transmission periods and heightening the potential for large-scale outbreaks [8]. Mosquito positive pools have exceeded 1000 annually from 2021 to 2024 (1117, 1555, 1146, and 1578, respectively), compared to 1024 in 2018. These years saw 135 human cases diagnosed (average 34/year) [18].

Beyond environmental factors, viral evolution remains a significant concern in WNV epidemiology. Genetic analyses suggest that WNV strains continue to evolve, potentially enhancing mosquito infectivity and transmission efficiency [9]. This evolution may contribute to the virus’s ability to persist in urban settings and influence disease severity in human hosts [9]. Certain genetic variants could be better suited for transmission by urban mosquito populations, partially explaining the continued outbreaks in NYC. However, debate remains regarding the extent to which genetic mutations impact transmission dynamics. While some researchers argue that evolving strains may lead to increased virulence or altered host preferences, others believe that environmental and ecological factors play a more substantial role. Future studies should prioritize genomic surveillance to assess whether emerging WNV variants exhibit higher virulence or vector competence. Understanding these evolutionary patterns will help refine public health strategies and anticipate shifts in disease transmission.

Incorporating climate-based risk assessments into vector surveillance and control programs is essential. The combined effects of climate change and viral evolution demand adaptive mosquito management strategies that account for shifting vector habitats, extended transmission periods, and the emergence of new viral strains. Proactive measures such as predictive modeling of outbreak patterns and early intervention strategies will be vital in mitigating future WNV risks in NYC and other metropolitan regions. While informative, correlation-based analyses such as Pearson coefficients have inherent limitations in capturing the full complexity of WNV transmission dynamics. Multiple interacting factors—including population density, mosquito species and feeding behavior, age structure, socioeconomic status, healthcare-seeking patterns, urban infrastructure, and micro-environmental conditions—can influence mosquito abundance and human case reporting. These bias-inducing factors may confound spatial associations, particularly in heterogeneous urban environments like New York City. Although our findings provide strong evidence of spatial and ecological relationships, a deeper understanding will require multivariate approaches that adjust for these interdependent influences. Future research should emphasize multivariable models and incorporate finer-scale demographic and environmental data to better define WNV risk and improve predictive modeling at the neighborhood level.

Ultimately, our findings underscore the need for a comprehensive, multidisciplinary approach to mitigating West Nile virus in urban environments. As city landscapes continue to evolve under the pressures of climate change and urbanization, integrating ecological, climatic, and genomic surveillance with targeted, data-driven mosquito control strategies will be essential to reducing WNV-related morbidity and mortality, particularly among high-risk populations.

## 5. Conclusions

The resurgence of WNV activity in New York City between 2010 and 2019 underscores the growing complexity of vector management in dense urban settings. While 2018 recorded the highest number of positive mosquito pools (1024), 2010 marked the peak in human cases (42), reflecting an ongoing challenge in mitigating transmission risk amid evolving mosquito behavior, changing land use, and climate variability [29].

This study confirms the significant and underrecognized role of *Cx. salinarius* in WNV transmission. Despite its lower abundance compared to *Cx. pipiens* and *Cx. restuans*, *Cx. salinarius* demonstrates heightened anthropophilic tendencies and a disproportionately strong association with human cases. These characteristics make it a critical target for vector control efforts. However, focused interventions alone may not suffice, as broader ecological factors—such as urban heat island effects and prolonged breeding seasons—continue to complicate disease prevention.

Our findings highlight the urgent need for an integrated, adaptive approach to WNV mitigation. Key strategies include targeted mosquito control, habitat modification, and reducing breeding sites, alongside robust public education and ongoing surveillance of vector populations. The concurrent rise of other arboviruses such as dengue and chikungunya [37,38] further underscores the necessity of strengthening vector-control infrastructure and maintaining readiness for future outbreaks.

As WNV evolves, genomic surveillance will become increasingly important for monitoring changes in viral transmissibility and virulence. A forward-looking, data-driven framework that combines ecological, climatic, and genetic surveillance with predictive modeling and early interventions will be essential to reducing WNV-related morbidity and mortality—both in New York City and in other at-risk metropolitan areas. These insights provide a foundation for scalable, science-based public health responses to vector-borne disease threats in the 21st century.

## Figures and Tables

**Table 1:** Surveillance of West Nile virus in human being and mosquitoes in NYC, 2000–2019.

Year	Human Cases Classification			Number of Positive Mosquito Pools				Pools Associated with Human Cases ***			
	Total Cases	Confirmed & Likely Local Cases *	Cases with Positive Mosquito Pools Within a 5-Mile Radius **	*Cx. pipiens* & *Cx. restuans*	*Cx. salinarius*	Other Species	Total Positive Pools	*Cx. pipiens* & *Cx. restuans*	*Cx. salinarius*	Other Species	Total Associated Positives Pools
2000	14	10	9	105	32	28	165	12	21	5	38
2001	9	5	3	210	24	9	243	9	0	0	9
2002	29	21	8	151	35	13	199	18	23	0	41
2003	32	25	22	234	35	8	277	40	33	0	73
2004	5	1	0	148	10	26	184	0	0	0	0
2005	14	13	9	119	3	0	122	17	3	0	20
2006	12	12	9	167	15	14	196	43	11	3	57
2007	18	17	4	159	12	3	174	9	0	0	9
2008	15	15	8	182	9	6	197	15	3	1	19
2009	3	2	0	39	1	0	40	0	0	0	0
2010	42	41	34	375	18	0	393	108	19	0	127
2011	11	11	7	170	9	2	181	11	9	1	21
2012	41	40	24	288	24	0	312	40	17	0	57
2013	10	9	3	236	11	2	249	6	1	2	9
2014	15	14	13	350	12	9	371	32	4	1	37
2015	38	32	26	789	32	6	827	158	31	3	192
2016	6	6	4	280	2	0	282	6	0	0	6
2017	21	20	13	759	24	3	786	49	9	0	58
2018	36	34	31	928	69	27	1024	119	53	11	183
2019	10	10	6	370	35	5	410	16	14	0	30
Total	381	338	233	6059	412	161	6632	708	251	27	986

* Human cases: Includes only those originating in NYC. Cases excluded are those with unknown or unlikely sources, no onset date, or no address. ** Mosquito pools: Includes all pools collected within 10 days prior to the onset dates of human cases. Each pool consists of groups of mosquitoes tested for WNV, with fewer than 50 mosquitoes per group. *** Spatial considerations: For *Cx. pipiens* and *Cx. restuans*, pools within a 2-mile radius were analyzed. For *Cx. salinarius*, pools within a 5-mile radius were included. For other species, a 2-mile radius was used for short-distance flyers, and a 5-mile radius for long-distance flyers.

**Table 2:** Analysis of mosquito species associated with human cases.

Characteristic	*Cx. pipiens* & *Cx. restuans*	*Cx. salinarius*	Other Species
Mosquito pools associated with human cases	708	251	27
Percentage of all positive pools for each species (%)	11.69%	60.92%	16.77%
Number of human cases associated	193	124	21
Human cases with only one positive mosquito species	106	38	2
Average distance to human cases (miles)	1.25	3.22	2.47
Percentage of mosquito pools associated with human cases within 1 mile (%)	22.21%	1.62%	0.61%
Percentage within 1–2 miles (%)	49.59%	2.33%	0.91%
Percentage within 2–5 miles (%)	0	21.5%	1.22%

## Data Availability

The data used in this study are part of ongoing public health surveillance activities conducted by the DOHMH and are not publicly available due to privacy and confidentiality considerations. Aggregate or summary data may be made available upon reasonable request and subject to applicable data sharing agreements and approvals.
